# Tricin Inhibits Human Osteosarcoma Cell Metastasis via Suppression of ZNF503 and the PI3K–PIP3–Akt Pathway

**DOI:** 10.1002/ddr.70374

**Published:** 2026-08-25

**Authors:** Chia‐Hsuan Chou, Jia‐Sin Yang, Pei‐Ni Chen, Yi‐Hsien Hsieh, Chih‐Hsin Tang, Ya‐Chiu Lin, Shun‐Fa Yang, Ko‐Hsiu Lu

**Affiliations:** ^1^ Department of Medical Research Chung Shan Medical University Hospital Taichung Taiwan; ^2^ Institute of Medicine Chung Shan Medical University Taichung Taiwan; ^3^ Department of Pharmacology, School of Medicine China Medical University Taichung Taiwan; ^4^ Department of Medical Laboratory Science and Biotechnology Asia University Taichung Taiwan; ^5^ Chinese Medicine Research Center China Medical University Taichung Taiwan; ^6^ Department of Orthopedics Chung Shan Medical University Hospital Taichung Taiwan; ^7^ School of Medicine Chung Shan Medical University Taichung Taiwan

**Keywords:** metastasis, osteosarcoma, PI3K–PIP3–Akt, tricin, ZNF503

## Abstract

Osteosarcoma is the most common primary malignant bone tumor and is associated with a high mortality rate due to its pronounced metastatic potential. Although tricin has been proposed as a chemosensitizer in various malignancies, its anti‐metastatic effect in osteosarcoma remains poorly characterized. This study investigated whether tricin suppresses metastasis in human osteosarcoma cells and explored the underlying molecular mechanisms. Our results revealed that tricin significantly inhibited the migration and invasion of U2OS and Saos‐2 osteosarcoma cells and downregulated ZNF503 expression at both the mRNA and protein levels. Similarly, siRNA‐mediated knockdown of ZNF503 suppressed cell migration, as confirmed by immunofluorescence analysis. Tricin treatment also reduced phosphorylation of PI3K and Akt, without affecting the activation of ERK1/2, JNK1/2, or p38. Additionally, intracellular levels of PIP3 were diminished following tricin exposure. Notably, the Akt activator SC‐79 significantly reversed tricin‐induced suppression of both ZNF503 expression and cell migration in U2OS and Saos‐2 cells. In conclusion, these findings suggest that ZNF503 inhibits osteosarcoma cell motility via the PI3K–PIP3–Akt signaling axis and that tricin exerts anti‐metastatic effects by targeting this pathway. Tricin may therefore represent a promising therapeutic agent for inhibiting osteosarcoma metastasis.

## 1 Introduction

Osteosarcoma is the most common primary malignant bone tumor, accounting for approximately 35% of all malignant bone tumors and 3%–5% of pediatric cancers (Lu et al. 2020, 2023; Mirabello et al. 2009; Picci et al. 2010). Its incidence peaks during adolescence (ages 10–15) and shows a secondary rise in older adults. Approximately 80%–90% of osteosarcomas originate in the metaphyseal regions of long bones, most frequently affecting the distal femur, proximal tibia, and proximal humerus (Lu et al. 2019, 2022, 2023). Its aggressive behavior, especially the high propensity for pulmonary metastasis, contributes to its poor prognosis and high mortality rate, positioning it among the most lethal pediatric malignancies (Cheng et al. 2016b; Lu et al. 2020). Historically, survival rates were below 20% when treatment was limited to surgical resection or amputation (Cheng et al. 2016a; Lu et al. 2018). The advent of neoadjuvant chemotherapy has significantly improved outcomes, increasing 5‐year survival rates to 60%–75% in patients with localized disease and allowing for limb‐salvage procedures in over 90% of cases (Lu et al. 2020, 2023). Nonetheless, the presence of lung metastases at diagnosis continues to reduce survival rates to approximately 20% (Lu et al. 2020, 2022, 2023; Oertel et al. 2010; Ottaviani and Jaffe 2009). Modern imaging modalities, such as PET/CT, PET/MRI, and dynamic MRI, have improved the detection of metastases and evaluation of treatment response (Casali et al. 2018; Liu et al. 2023b; Yang et al. 2024). Meanwhile, novel therapeutic strategies are being investigated to overcome treatment resistance and improve clinical outcomes.

Metastasis is a highly complex and tightly regulated multistep process, collectively referred to as the metastatic cascade (Reiter et al. 2017; Su et al. 2017). This sequence involves epithelial–mesenchymal transition (EMT), detachment from the primary tumor, extracellular matrix (ECM) degradation, invasion and migration, intravasation into the vasculature, survival in circulation, extravasation, mesenchymal–epithelial transition, and colonization at distant sites (Gupta and Massague 2006; Meyer and Hart 1998). Metastasis, driven by the acquisition of aggressive phenotypes that promote dissemination and secondary tumor formation, is responsible for 90% of cancer patient deaths (Fidler et al. 2007; Sheng et al. 2009). Throughout this process, cancer cells must evade apoptosis, maintain proliferative and migratory capacity, adapt to new microenvironments, and induce angiogenesis (Sheng et al. 2009). Simultaneously, they face numerous obstacles, including immune surveillance, loss of adhesion, integrin dysfunction, hypoxia, nutrient deprivation, and mechanical stress during transendothelial migration.

Among the major signaling pathways involved in metastasis, the mitogen‐activated protein kinases (MAPKs) and the phosphoinositide 3‐kinase (PI3K)–Akt pathway are key regulators activated by diverse extracellular stimuli (Reddy et al. 2003). These cascades control essential cellular processes such as motility, invasion, ECM degradation, and angiogenesis. Specifically, the extracellular signal‐regulated protein kinase (ERK)1/2 and c‐Jun N‐terminal kinase (JNK) pathways have been implicated in the regulation of matrix metalloproteinases (MMPs), which facilitate ECM remodeling and tumor cell migration (Chen et al. 2011; Hsieh et al. 2007; Reddy et al. 2003). In osteosarcoma cells, p38 and Akt signaling have been shown to suppress MMP‐2 and MMP‐9 expression, thereby modulating invasiveness (Yang et al. 2013). Among these, Akt serves as a central node that regulates numerous downstream targets involved in cell survival, proliferation, EMT, motility, and metastasis upon phosphorylation‐mediated activation (Sheng et al. 2009).

Tricin, a naturally occurring flavone, exhibits multiple biological activities, including antioxidant, antiviral (anti‐influenza), and hepatoprotective effects (Moheb et al. 2013). Flavonoids, including tricin, have demonstrated antitumor properties through mechanisms such as inhibition of proliferation, induction of apoptosis, suppression of protein tyrosine kinase activity, and antiangiogenic effects (Middleton et al. 2000). Despite its favorable pharmacokinetic profile and potential as a chemopreventive agent, the anticancer and, particularly, the antimetastatic potential of tricin remains inadequately studied. While tricin has been proposed as a promising therapeutic agent in oncology (Cai et al. 2004; Hudson et al. 2000; Oyama et al. 2009), its effects on human osteosarcoma metastasis remain to be fully elucidated. In this study, we investigated the in vitro antimetastatic effects of tricin on human osteosarcoma U2OS and Saos‐2 cells and explored the underlying molecular mechanisms involved in its action.

## 2 Materials and Methods

### 2.1 Materials

Dulbecco's modified Eagle medium (DMEM), RPMI 1640 medium (RPMI), penicillin–streptomycin, and fetal bovine serum (FBS) were obtained from Gibco‐BRL (Gaithersburg, MD, USA) and Hyclone Laboratories, Inc. (Logan, UT, USA), respectively. SC‐79, antibodies against β‐actin and zinc finger protein 503 (ZNF503) were sourced from Abcam (Cambridge, UK). Antibodies against phosphorylated (p‐) and total forms of ERK1/2 and JNK1/2, as well as p‐Akt (Ser473), were purchased from Cell Signaling Technology (Danvers, MA, USA). Antibodies targeting PI3K and Akt were obtained from BD Biosciences (Bedford, MA, USA). Antibodies against PI3K p85α, PI3K p110, total p38, and p‐p38 were obtained from Santa Cruz Biotechnology (Dallas, TX, USA). Antibodies specific for phosphatidylinositol 3,4,5‐trisphosphate (PIP3) were purchased from Invitrogen (Waltham, MA, USA). Small interfering RNA (siRNA) targeting ZNF503 was acquired from Ambion (Austin, TX, USA).

### 2.2 Cell Culture and Tricin Treatment

**Figure 1:**
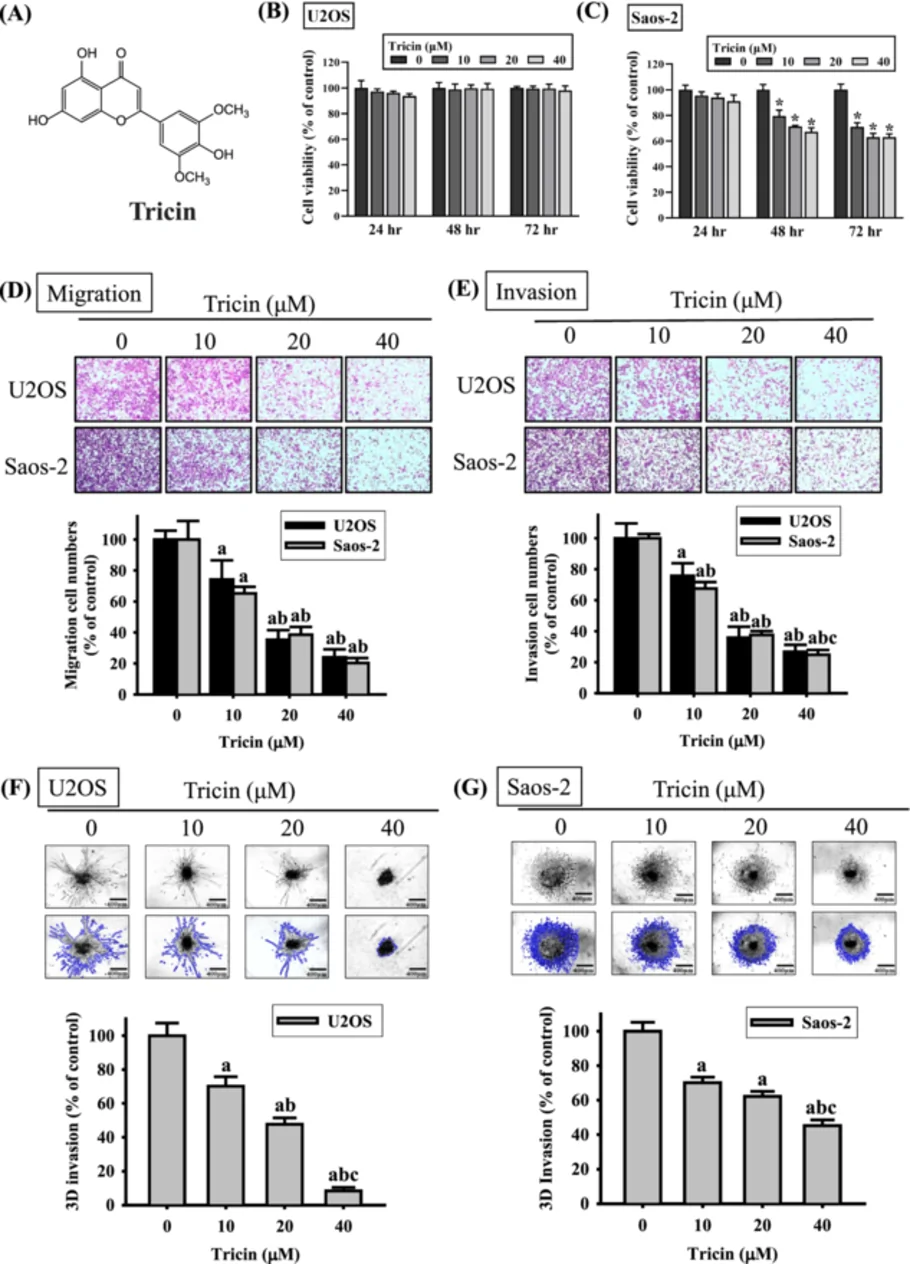
Structure of tricin and its effects on cell viability, cell migration, invasion, and 3D tumor invasion in U2OS and Saos‐2 cells. (A) Chemical structure of the flavonoid tricin (4′,5,7‐trihydroxy‐3′,5′‐dimethoxyflavone). (B, C) Cell viability of U2OS and Saos‐2 cells treated with tricin at concentrations of 0, 10, 20, and 40 μM for 24, 48, and 72 h was assessed using the MTT assay. Data are presented as mean ± SD; *p < 0.05. (D) Migration and (E) invasion assays were performed on U2OS and Saos‐2 cells treated with tricin (0, 10, 20, and 40 μM) for 24 h. (F, G) 3D tumor invasion assays were conducted on U2OS and Saos‐2 cells following treatment with tricin (0, 10, 20, and 40 μM) for 72 h. Data are presented as mean ± SD (*n* = 3). Statistical analysis was performed using one‐way ANOVA with Tukey's post hoc test. Migration: U2OS: *F* = 59.477, *p* < 0.001; Saos‐2: *F* = 72.879, *p* < 0.001. Invasion: U2OS: *F* = 62.551, *p* < 0.001; Saos‐2: *F* = 333.387, *p* < 0.001. 3D tumor invasion (*n* = 4): U2OS: *F* = 56.255, *p* < 0.001; Saos‐2: *F* = 37.106, *p* < 0.001. ^a^Significantly different, *p* < 0.05, when compared to control. ^b^Significantly different, *p* < 0.05, when compared to 10 μM. ^c^Significantly different, *p* < 0.05, when compared to 20 μM.

Human osteosarcoma cell lines U2OS (derived from a 15‐year‐old female) and Saos‐2 (from 11‐year‐old Caucasian female) were obtained from the Food Industry Research and Development Institute (Hsinchu, Taiwan) and the American Type Culture Collection (ATCC, Manassas, VA, USA), respectively. Cells were cultured in DMEM supplemented with 10% FBS and 1% penicillin/streptomycin and maintained in a humidified incubator at 37°C with 5% CO2. Tricin was purchased from ChromaDex (Longmont, CO, USA) (Figure 1A).

### 2.3 Microculture Tetrazolium (MTT) Assay

Cell viability following tricin treatment was assessed using a colorimetric MTT assay (3‐(4,5‐dimethylthiazol‐2‐yl)‐2,5‐diphenyltetrazolium bromide). U2OS (8.5 × 10^4^/well) and Saos‐2 (1.0 × 10^5^/well) cells were seeded in 24‐well plates, incubated for 16 h, and then treated with tricin (0, 10, 20, and 40 μM) at 37°C for 24 h. After treatment, MTT solution (0.5 mg/mL) was added and incubated for 4 h at 37°C, followed by solubilization and absorbance measurement. The assay was performed as previously described (Hsieh et al. 2007; Lu et al. 2013).

### 2.4 Migration and Invasion Assays

Cell motility was evaluated using Boyden chamber assays. For invasion assays, chambers were pre‐coated with Matrigel. Following tricin treatment (0, 10, 20, and 40 μM), U2OS (1.0 × 10^4^/well) and Saos‐2 (1.25 × 10^4^/well) cells were seeded in the upper chambers (Neuro Probe, Cabin John, MD, USA) and incubated at 37°C for 24 h. Migrated or invaded cells were fixed, stained, and quantified under a light microscope (Hsieh et al. 2007; Lu et al. 2013). To better mimic the native tumor environment, a three‐dimensional (3D) tumor invasion assay was also conducted.

### 2.5 3D Tumor Invasion Assay

U2OS and Saos‐2 spheroids were generated by seeding 1,000 cells per well in 96‐well ultra‐low attachment (ULA) plates (Cat. #7007, Corning, NY, USA) and incubating for 24–72 h. Spheroids were then embedded in 2 mg/mL type I collagen (Cat. #354249, Corning). Collagen stock (9.6 mg/mL) was neutralized with 1 N NaOH (Sigma‐Aldrich) and 5× DMEM to achieve a final pH of ~7.4. A 100 μL aliquot of the matrix containing spheroids was added per well and polymerized at 37°C for 1 h, followed by the addition of 100 μL of complete medium. Spheroids were treated with tricin (dissolved in DMSO) or 0.1% DMSO as a control. Invasion was assessed on Day 3 using ImageJ software (National Institutes of Health) by calculating the invaded area (total area minus spheroid core area).

### 2.6 Western Blot Analysis

To evaluate protein expression, U2OS (7.5 × 10^5^) and Saos‐2 (9.0 × 10^5^) cells were seeded in 6‐cm dishes, incubated for 16 h, and treated with tricin (0, 10, 10, and 40 μM) for 24 h. Total cell lysates were prepared as previously described (Hsieh et al. 2007; Lu et al. 2016, 2013), separated by sodium dodecyl sulfate–polyacrylamide gel electrophoresis (SDS‐PAGE), and transferred to membranes. Membranes were probed with primary antibodies against ZNF503 and total and phosphorylated forms of PI3K (p85), PI3K (p110), Akt (Ser473), and MAPK pathway proteins (ERK 1/2, JNK 1/2, and p38). Detection was performed using horseradish peroxidase (HRP)‐conjugated secondary antibodies, and signals were quantified by densitometry (Hsieh et al. 2007; Lu et al. 2016, 2013).

### 2.7 RT‐PCR and Real‐Time PCR

U2OS (2.4 × 10^5^) and Ssos‐2 (4.5 × 10^5^) cells were seeded in 6‐well, incubated for 16 h, and treated with tricin (0, 10, 20, and 40 μM) for 24 h. Total RNA was extracted using the Total RNA Mini Kit (Geneaid, New Taipei City, Taiwan), and complementary DNA (cDNA) was synthesized using the High‐Capacity cDNA Reverse Transcription Kit (Applied Biosystems, CA, USA). RT‐PCR was performed using primers for ZNF503 and glyceraldehyde‐3‐phosphate dehydrogenase (GAPDH) as described previously (Hsieh et al. 2007, 2004). Quantitative real‐time PCR was performed in triplicate using the StepOnePlus system, with threshold cycles analyzed within the linear amplification range (Lu et al. 2013).

### 2.8 RNA Sequencing and Gene Expression Analysis

U2OS (2.4 × 10^5^) and Saos‐2 (4.5 × 10^5^) cells were seeded in 6‐well plates, incubated for 16 h, and treated with 40 μM tricin for 24 h. RNA sequencing (RNA‐seq) was outsourced to BIOTOOLS. Differentially expressed genes (fold change > 2) were analyzed and visualized via hierarchical clustering heat maps (Lu et al. 2018).

### 2.9 Small‐Interfering RNA (SiRNA) Transfection

ZNF503 was silenced using siRNA (Applied Biosystems, Foster City, CA, USA). The ZNF503 siRNA sequences were as follows: sense, 5′‐GCGCAAACAUGUUCGCAGATT‐3′, and antisense, 5′‐UCUGCGAACAUGUUUGCGCCA‐3′; U2OS (7 × 10^5^) and Soas‐2 (9 × 10^5^) cells were seeded in 6‐cm dishes and transfected with 150 pmol of ZNF503 siRNA using Lipofectamine RNAiMAX (Invitrogen, Carlsbad, CA, USA) according to the manufacturer's protocol. A nonspecific siRNA duplex was used as the negative control (Lu et al. 2018).

### 2.10 Immunofluorescence Analysis

Cells treated with tricin (0, 10, 20, and 40 μM) were cultured on glass coverslips, fixed in 4% paraformaldehyde for 20 min, and permeabilized with 0.5% Triton X‐100. After washing with PBS, cells were blocked with 5% bovine serum albumin for 1 h and incubated overnight at 4°C with antibodies against ZNF503 (1:50) or PIP3 (1:100). Cells were then washed and incubated with fluorescein isothiocyanate (FITC)‐conjugated secondary antibodies for 1 h at room temperature. Nuclei were counterstained with 4′,6‐diamidino‐2‐phenylindole (DAPI) for 5 min, and coverslips were mounted using DAKO mounting medium Glostrup, Denmark). Imaging was captured using a Zeiss LSM 510 META confocal microscope (Lu et al. 2018).

### 2.11 Statistical Analysis

Data are presented as mean ± standard deviation (SD) from three independent experiments, each performed in triplicate. Statistical significance was evaluated using one‐way analysis of variance (ANOVA) for more than two groups, followed by Scheffé's post hoc test for unequal group sizes or Tukey's test for equal group sizes. For comparisons between two groups, Student's *t*‐test was used. A *p*‐value < 0.05 was considered statistically significant.

## 3 Results

### 3.1 Cytotoxicity of Tricin in Osteosarcoma U2OS and Saos‐2 Cells

The cytotoxic effects of tricin were evaluated in human osteosarcoma U2OS and Saos‐2 cell lines using the MTT assay. Following 24, 48, and 72 h of treatment, tricin at concentrations of 10, 20, and 40 μM did not significantly affect the viability of U2OS cells compared with the untreated control group (0 μM) at any time point (Figure 1B). In contrast, while no significant effect was observed in SAOS‐2 cells after 24 h of treatment, tricin induced a slight but significant reduction in cell viability after 48 and 72 h (*p* < 0.05), suggesting a time‐dependent inhibitory effect in this cell line (Figure 1C). These findings indicate that tricin, at concentrations up to 40 μM, does not exert cytotoxic effects over a 24‐h exposure period. Accordingly, concentrations up to 40 μM were employed in all subsequent experiments to assess the anti‐metastatic properties of tricin.

### 3.2 Tricin Inhibits Migration, Invasion, and 3D Invasion of U2OS and Saos‐2 Cells

The anti‐metastatic properties of tricin were further evaluated using Boyden chamber‐based migration and invasion assays. Following 24 h of treatment, tricin significantly inhibited the migratory capacity of U2OS (*p* < 0.001) and Saos‐2 (*p* < 0.001) cells in a concentration‐dependent manner (Figure 1D). Similarly, tricin markedly suppressed the invasive behavior of both cell lines when assessed using Matrigel‐coated Boyden chambers (U2OS: *p* < 0.001; Saos‐2: *p* < 0.001) (Figure 1E). To better replicate the complexity of the tumor microenvironment, a 3D tumor invasion assay was performed. As shown in Figure 1F,G, tricin significantly reduced the invasive capability of U2OS and Saos‐2 cells concentration‐dependently in the 3D model as well (U2OS: *p* < 0.001; Saos‐2: *p* < 0.001) (Figure 1F,G).

### 3.3 Tricin Downregulates ZNF503 Expression in U2OS and Saos‐2 Cells

**Figure 2:**
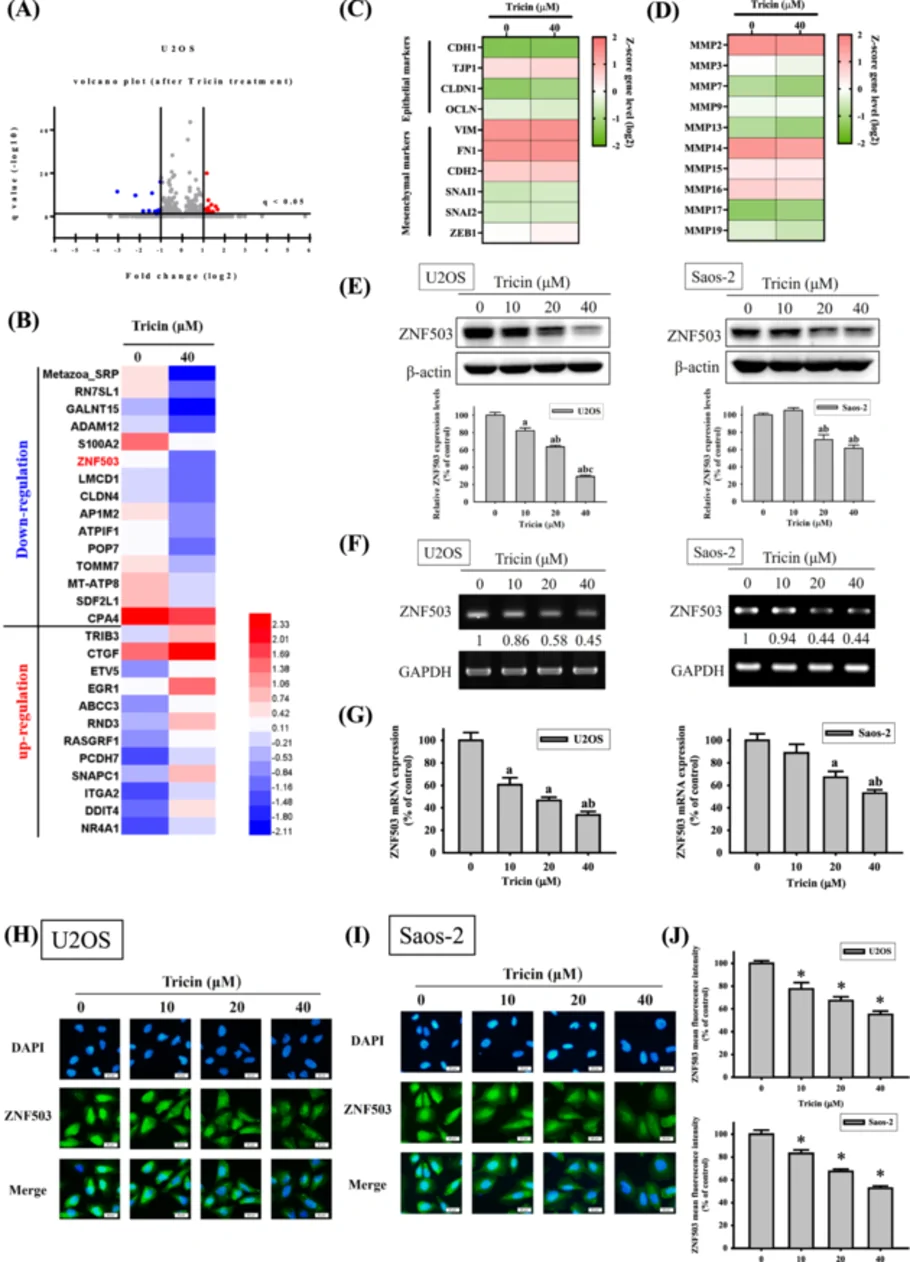
ZNF503 expression in tricin‐treated U2OS and Saos‐2 cells. (A) Volcano plot and (B) Heatmap showing gene expression profiles in U2OS cells treated with or without 40 μM tricin for 24 h. Two‐way hierarchical clustering was applied; red indicates upregulation, and blue indicates downregulation. Notably, ZNF503 expression was markedly suppressed. (C) Heatmap showing EMT‐related markers gene expression profiles in U2OS cells treated with or without 40 μM tricin for 24 h. (D) Heatmap showing MMP family members gene expression profiles in U2OS cells treated with or without 40 μM tricin for 24 h. (E) Western blot analysis of ZNF503 in U2OS and Saos‐2 cells treated with increasing concentrations of tricin (0–40 μM) for 24 h. (F) RT‐PCR and (G) real‐time PCR analyses of ZNF503 were conducted in U2OS and Saos‐2 cells treated with tricin (0–40 μM) for 24 h. Data are shown as mean ± SD. Statistical analysis was performed using ANOVA, followed by Tukey's test for Western blotting and RT‐PCR. (*n* = 3). Western blotting: U2OS: *F* = 131.924, *p* < 0.001; Saos‐2: *F* = 31.235, *p* < 0.001. Statistical significance was determined using ANOVA with Scheffé's test for real‐time PCR. U2OS (*n* ≥ 5): *F* = 29.652, *p* < 0.001; Saos‐2 (*n* ≥ 4): *F* = 14.575, *p* < 0.001. ^a^Significantly different, *p* < 0.05, when compared to control. ^b^Significantly different, *p* < 0.05, when compared to 10 μM. ^c^Significantly different, *p* < 0.05, when compared to 20 μM. (H, I) Immunofluorescent staining revealed a concentration‐dependent reduction in ZNF503 expression following treatment with 0, 10, 20, and 40 μM of tricin for 24 h in U2OS and Saos‐2 cells. (J) Quantitative analysis of ZNF503 fluorescence intensity is shown. *p < 0.05, compared with vehicle control using Student's *t*‐test.

To elucidate the molecular basis underlying the anti‐metastatic effects of tricin, transcriptomic profiling was performed via RNA sequencing on U2OS cells treated with or without 40 μM tricin for 24 h. A total of 27 differentially expressed genes (DEGs) were identified, including 15 downregulated and 12 upregulated genes with a fold change greater than two (Figure 2A). The top 27 genes that were most significantly upregulated and downregulated in response to tricin treatment were annotated and visualized in the form of a heat map (Figure 2B). Among the downregulated genes, ZNF503 was selected for further investigation because it exhibited marked suppression following tricin treatment and has been reported to function as a transcription factor involved in cancer progression, particularly in regulating cell migration and invasion (Shahi et al. 2015). However, the expression levels of EMT‐related markers (Figure 2C) and metastasis‐associated genes, including MMP family members (Figure 2D), showed no significant changes following tricin treatment. These transcriptomic findings were validated by Western blot, RT‐PCR, and quantitative real‐time PCR, all of which confirmed that treatment with 40 μM tricin significantly reduced ZNF503 expression at both the mRNA and protein levels in U2OS and Saos‐2 cells (U2OS: Western: *p* < 0.001, RT‐PCR: *p* < 0.001, real‐time PCR: *p* < 0.001; Saos‐2: Western: *p* < 0.001, RT‐PCR: *p* < 0.001, real‐time PCR: *p* < 0.001) (Figure 2E,G). Furthermore, immunofluorescence staining demonstrated a marked reduction in ZNF503 protein expression with increasing concentrations of tricin in both osteosarcoma cell lines (Figure 2H–J).

### 3.4 ZNF503 Knockdown Inhibits Migration in U2OS and Saos‐2 Cells

**Figure 3:**
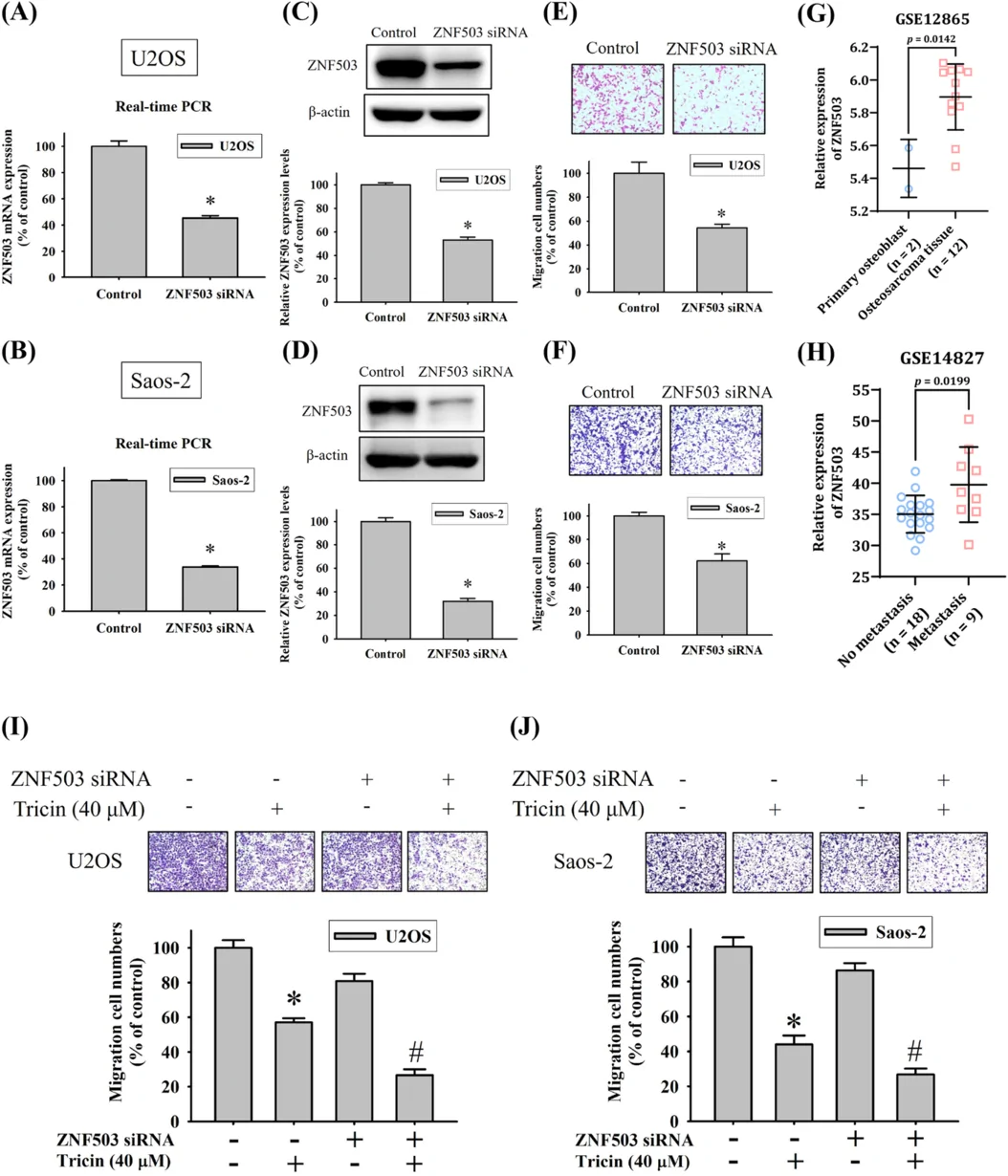
Effects of ZNF503 knockdown on migration and invasion in U2OS and Saos‐2 cells. (A, B) Real‐time PCR and (C, D) Western blot analysis of ZNF503 expression following transfection with ZNF503 siRNA for 48 h. (E, F) After 24 h, migration assays were used to assess the functional impact of ZNF503 silencing. Data are shown as mean ± SD (*n* = 3). Student's *t*‐test was used for statistical analysis. Real‐time PCR: U2OS: *p* = 0.002; Saos‐2: *p* < 0.001. Western blotting: U2OS: *p* < 0.001; Saos‐2: *p* < 0.001. Migration: U2OS: *p* = 0.028; Saos‐2: *p* = 0.011. *p < 0.05. (G) ZNF503 mRNA levels in control normal tissues and osteosarcoma tissues. (H) Effect of ZNF503 mRNA levels on pulmonary metastasis of osteosarcoma tissues. (I, J) Human U2OS and Saos‐2 cell lines were pre‐treated with ZNF503 siRNA for 24 h and then incubated in the presence or absence of tricin (40 μM) for 24 h and the migration ability of cells were observed by Boyden chamber assay. *p < 0.05, compared with the vehicle group. ^#^ *p* < 0.05, compared with the tricin treated group.

To further assess the functional role of ZNF503 in cell migration, U2OS and Saos‐2 cells were transfected with ZNF503‐specific siRNA. After 48 h post‐transfection, both mRNA and protein levels of ZNF503 were significantly reduced in U2OS and Soas‐2 cells, as confirmed by real‐time PCR and Western blot analyses (U2OS: mRNA: *p* = 0.002, protein: *p* < 0.001; Saos‐2: mRNA: *p* < 0.001, protein: *p* < 0.001) (Figure 3A–D). Consistent with the observed molecular changes, Boyden chamber migration assays revealed that ZNF503 knockdown significantly impaired the migratory ability of U2OS (*p* = 0.028) and Saos‐2 (*p* = 0.011) cells (Figure 3E,F). Additionally, analysis of the GEO dataset confirmed that ZNF503 mRNA levels are significantly elevated in osteosarcoma tissues (Figure 3G) and are associated with pulmonary metastasis (Figure 3H). Moreover, to further investigate the involvement of ZNF503 in the anti‐migratory effect of tricin, U2OS and Saos‐2 cells were treated with ZNF503 siRNA in combination with tricin (40 μM). As shown in Figure 3I–J, tricin treatment alone significantly suppressed cell migration in both cell lines. Furthermore, co‐treatment with ZNF503 siRNA and tricin resulted in a more pronounced reduction in migratory ability than either treatment alone. These results indicate that ZNF503 depletion enhances the inhibitory effect of tricin on osteosarcoma cell migration, suggesting that ZNF503 contributes to the regulation of migratory behavior and may be involved in the anti‐metastatic mechanism of tricin.

### 3.5 Tricin Decreases ZNF503 Expression to Inhibit Cell Migration via the PI3K–PIP3–Akt Pathway in U2OS and Saos‐2 Cells

**Figure 4:**
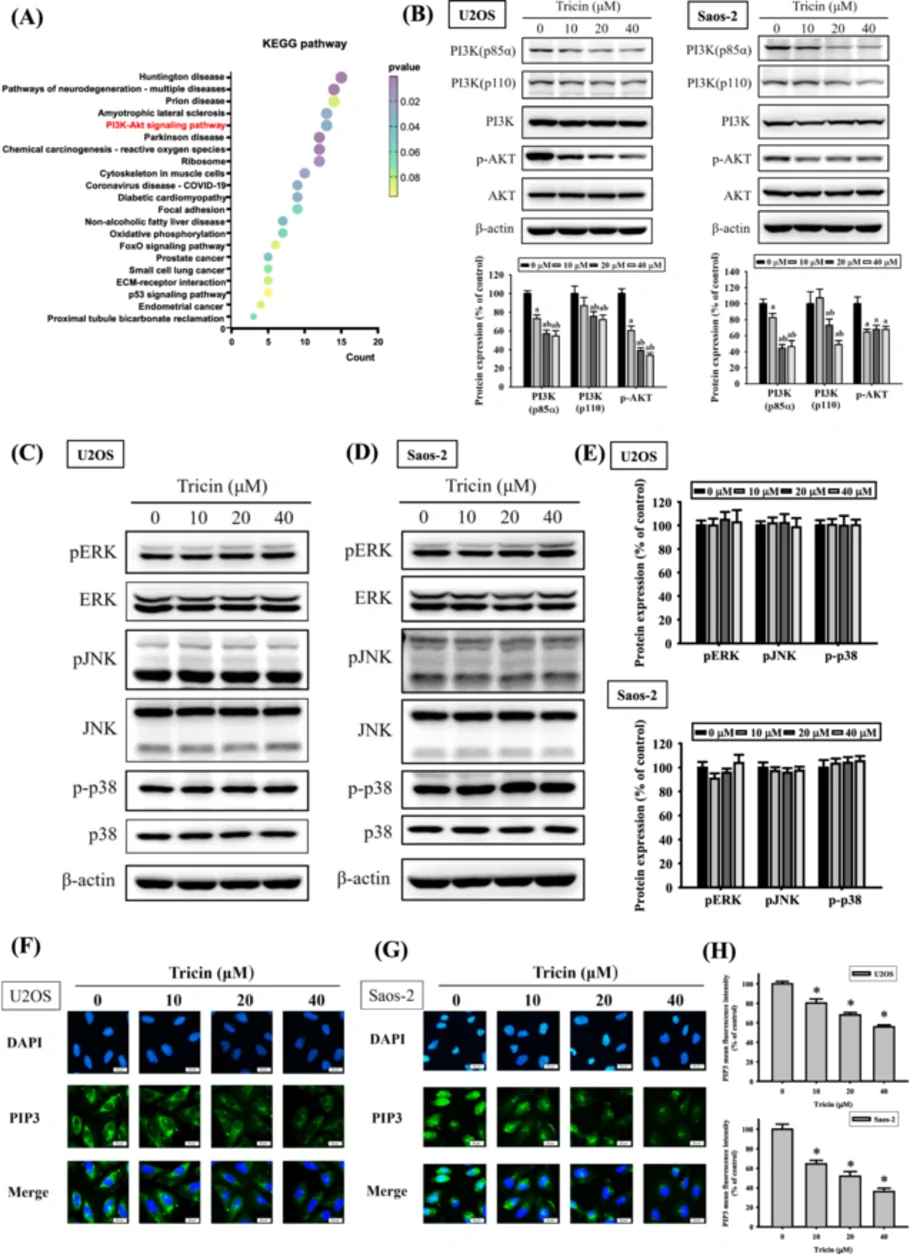
Effects of tricin on PI3K–Akt, and MAPK signaling in U2OS and Saos‐2 cells. (A) Schematic representation of the KEGG signaling pathway affected by tricin. (B) Western blot analyses showing total and phosphorylated levels of PI3K (p85), PI3K (p110), and Akt in U2OS and Saos‐2 cells treated with tricin (0–40 μM). (C–E) Western blot analyses showing total and phosphorylated levels of ERK1/2, JNK1/2, and p38 in U2OS and Saos‐2 cells treated with tricin (0–40 μM). Data are presented as mean ± SD (*n* = 3). One‐way ANOVA with Tukey's post hoc test was used. PI3K (p85): U2OS: *F* = 72.402, *p* < 0.001; Saos‐2: *F* = 64.998, *p* < 0.001. Akt: U2OS: *F* = 162.568, *p* < 0.001; Saos‐2: *F* = 26.840, *p* < 0.001; ERK1/2: U2OS: *F* = 0.314, *p* = 0.815; Saos‐2: *F* = 3.817, *p* = 0.058. JNK1/2: U2OS: *F* = 0.221, *p* = 0.879; Saos‐2: *F* = 0.700, *p* = 0.578. p38: U2OS: *F* = 0.006, *p* = 0.999; Saos‐2: *F* = 0.601, *p* = 0.633. ^a^Significantly different, *p* < 0.05, when compared to control. ^b^Significantly different, *p* < 0.05, when compared to 10 μM. (F, G) PIP3 expression was concentration‐dependently reduced in both U2OS and Saos‐2 cells after 24‐h tricin treatment (0, 10, 20, and 40 μM), as revealed by immunofluorescent staining. (H) Quantitative analysis of PIP3 fluorescence intensity is shown. *p < 0.05, compared with vehicle control using Student's *t*‐test.

To elucidate the upstream signaling mechanisms through which tricin modulates ZNF503 expression and suppresses cell migration, KEGG pathway enrichment mapping of transcriptomic alterations, was performed (Figure 4A). Moreover, western blot analysis was conducted to examine the activation status of key signaling pathways, including PI3K–Akt, and MAPKs (ERK1/2, JNK1/2, and p38) (Figure 4B–E). Treatment with tricin for 6 h resulted in a concentration‐dependent decrease in the phosphorylation of PI3K (p85) (U2OS: *p* < 0.001; Saos‐2: *p* < 0.001), PI3K (p110) (U2OS: *p* < 0.001; Saos‐2: *p* < 0.001) and Akt (Ser473) (U2OS: *p* < 0.001; Saos‐2: *p* < 0.001), while total protein levels remained unchanged (Figure 4B). No significant changes were observed in the phosphorylation or total protein levels of ERK1/2 (U2OS: *p* = 0.815; Saos‐2: *p* = 0.058), JNK1/2 (U2OS: *p* = 0.879; Saos‐2: *p* = 0.578), or p38 (U2OS: *p* = 0.999; Saos‐2: *p* = 0.633) (Figure 4C–E). Furthermore, immunofluorescence staining revealed a marked, concentration‐dependent reduction in intracellular levels of PIP3 following tricin treatment at 0, 10, 20, and 40 μM in both U2OS and Saos‐2 cells (Figure 4F–H).

**Figure 5:**
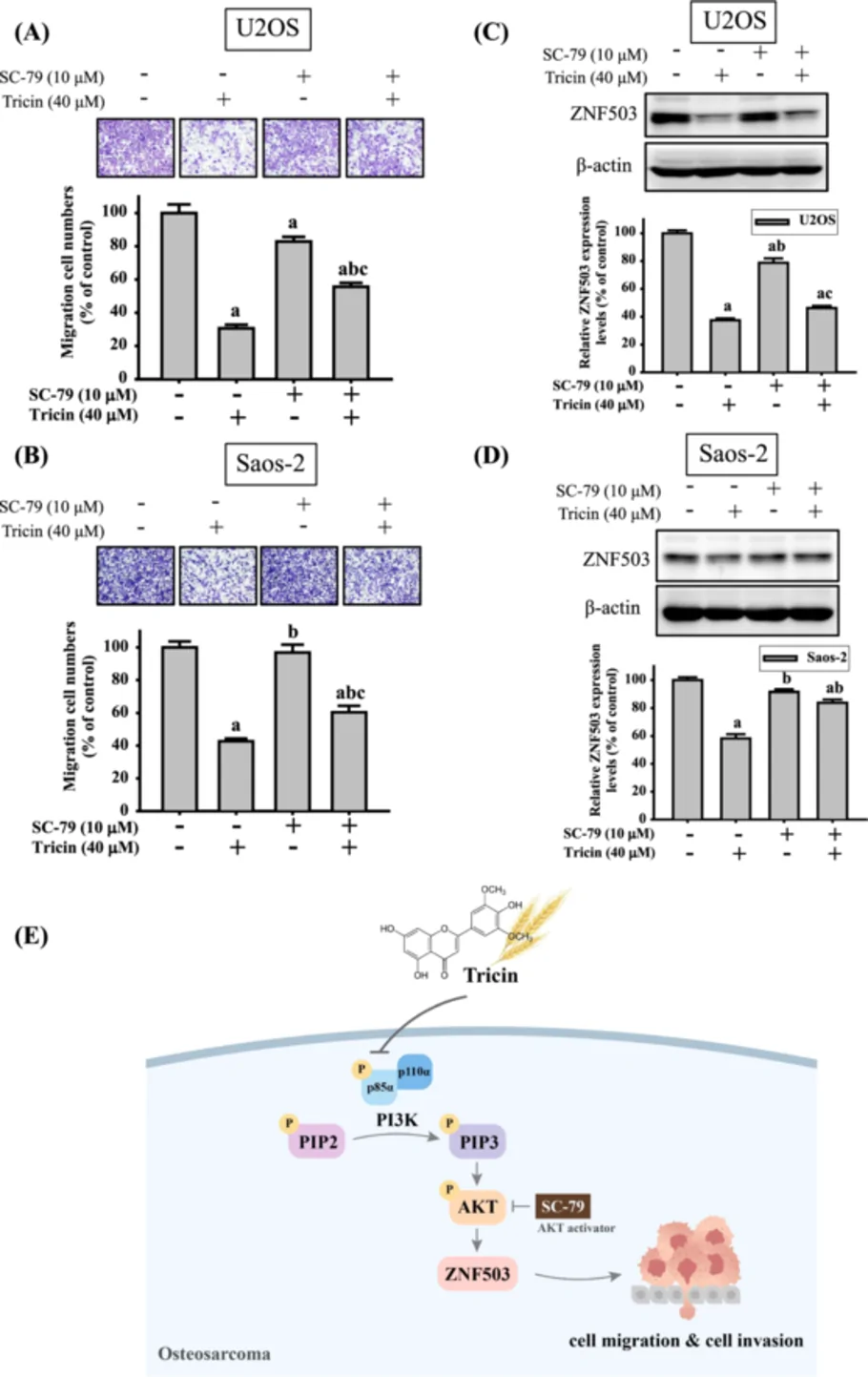
Effects of the Akt activator SC‐97 on tricin‐induced suppression of migration and ZNF503 expression in U2OS and Saos‐2 cells. (A, B) Migratory assays were performed using Boyden chamber assays to evaluate the migratory capability of U2OS and Saos‐2 cells treated with tricin (0 and 40 μM) for 24 h, with or without 10 μM of SC‐79. (C, D) Western blot analyses of ZNF503 expression in U2OS and Saos‐2 cells under the same treatment conditions. Data are shown as mean ± SD (*n* = 3). Statistical analysis was performed using ANOVA, followed by Tukey's test. U2OS: migration: F = 80.711, *p* < 0.001; ZNF503: F = 176.086, *p* < 0.001. Saos‐2: migration: F = 57.515, *p* < 0.001; ZNF503: F = 59.571, *p* < 0.001. ^a^Significantly different, *p* < 0.05, compared with control. ^b^Significantly different, *p* < 0.05, compared with 40 μM of tricin. ^c^Significantly different, *p* < 0.05, compared with 10 μM of SC‐79. (E) A schematic diagram illustrates that tricin suppresses the invasive and migratory behavior of U2OS and Saos‐2 osteosarcoma cells by inhibiting ZNF503 expression and the PI3K–PIP3–Akt pathway.

To further determine whether the suppression of PIP3 and PI3K–Akt phosphorylation by tricin mediates its inhibitory effects on ZNF503 expression and cell migration, the Akt activator SC‐79 was employed. As shown in Figure 5A–D, cotreatment with 10 μM SC‐79, with or without 40 μM tricin, modulated both ZNF503 expression and migratory capacity. Tricin alone significantly suppressed cell migration (*p* < 0.05) and ZNF503 expression (*p* < 0.05), while SC‐79 treatment alone induced their action (migration: *p* < 0.05; ZNF503: *p* < 0.05). Notably, SC‐79 significantly reversed the inhibitory effects of tricin on both migration and ZNF503 expression in U2OS and Saos‐2 cells (U2OS: migration: *p* < 0.05, ZNF503: *p* < 0.05; Saos‐2: migration: *p* < 0.05, ZNF503: *p* < 0.05). Collectively, these results strongly indicate that tricin exerts its anti‐metastatic effects predominantly through inhibition of the PI3K–PIP3–Akt signaling axis, leading to downregulation of ZNF503 and suppression of osteosarcoma cell migration.

## 4 Discussion

This study demonstrated that tricin, at non‐cytotoxic concentrations (≤ 40 μM), significantly inhibited the migration and invasion of human osteosarcoma U2OS and Saos‐2 cells. Tricin treatment downregulated ZNF503 at both the mRNA and protein levels, and siRNA‐mediated silencing of ZNF503 recapitulated the anti‐migratory phenotype. Moreover, tricin attenuated phosphorylation of PI3K (p85), PI3K (p110), and Akt, without affecting the expression or phosphorylation of ERK1/2, JNK1/2, or p38 MAPK. Consistently, immunofluorescence revealed a concentration‐dependent reduction in intracellular PIP3. Importantly, the Akt activator SC‐79 reversed tricin‐induced suppression of both ZNF503 expression and cell migration, indicating that tricin acts primarily through inhibition of the PI3K–PIP3–Akt axis rather than through MAPK signaling.

Tricin, a flavonoid abundant in the bran layers of cereals such as wheat and rice (Goufo and Trindade 2014), has attracted attention for its biological activities. Although wheat hulls and rice bran are not common dietary staples in many regions, rice‐bran derivatives (e.g., *Oryzae Fructus Germinatus*) are widely used in traditional Chinese medicine (Moheb et al. 2013; Poulev et al. 2018). As a secondary plant metabolite, tricin contributes to plant defense against pathogens, herbivores, and environmental stressors (Zhou and Ibrahim 2010) and exists in both free and glycosylated forms across a variety of cereal and grasses (Zhou and Ibrahim 2010). Previous studies have demonstrated tricin's antiproliferative, chemopreventive, antioxidant, and anti‐inflammatory properties, particularly in gastrointestinal and breast cancer models (Cai et al. 2004; Hudson et al. 2000; Kandaswami et al. 2005; Oyama et al. 2009). Despite these advances, the role of tricin in osteosarcoma remains unclear. In this study, we demonstrate that tricin suppresses osteosarcoma cell migration, thereby extending the biological relevance of tricin and suggesting its potential as a therapeutic candidate for osteosarcoma intervention.

ZNF503 has emerged as a driver of tumor aggressiveness in breast cancer, hepatocellular carcinoma, and non‐small cell lung cancer by enhancing migration and invasion (Liu et al. 2023a; Qi and Li 2020; Yin et al. 2019). Our findings extend this paradigm to osteosarcoma, demonstrating that ZNF503 regulates metastatic traits in this context as well. Notably, suppression of ZNF503 by tricin is associated with reduced migratory capacity of osteosarcoma cells, suggesting that ZNF503 may serve as a critical modulator of tumor cell dissemination. These results provide new insight into the functional relevance of ZNF503 in osteosarcoma and support its potential as a therapeutic target in limiting disease progression.

Unlike the MAPK cascade, which remained unaffected by tricin, the phosphatase and tensin homolog deleted on chromosome 10 (PTEN)–PI3K–Akt pathway was markedly suppressed. PI3K phosphorylates phosphatidylinositol‐4,5‐bisphosphate (PIP2) to generate PIP3, which recruits Akt to the membrane for activation (Carnero et al. 2008; Sheng et al. 2009), whereas PTEN opposes this step by dephosphorylating PIP3. The observed reduction in PIP3, along with the ability of SC‐79 to rescue ZNF503 expression and migratory potential, supports the conclusion that tricin targets this upstream signaling hub to exert its antimetastatic effects. However, the precise mechanism underlying tricin‐mediated regulation of protein expression remains to be determined whether this effect involves proteasomal degradation, translational suppression, or autophagy/lysosomal pathways warrants further investigation in future studies.

In conclusion, ZNF503 inhibits the invasive and migratory behavior of U2OS and Saos‐2 osteosarcoma cells by inactivating the PI3K–PIP3–Akt pathway, independently of MAPK signaling (Figure 5E). Tricin potently suppresses ZNF503 and inhibits this axis, thereby impairing osteosarcoma cell metastasis through a mechanism distinct from classical signaling pathways. These findings highlight tricin's therapeutic potential and warrant further investigation into its pharmacodynamics, bioavailability, and efficacy in in vivo metastasis models.

## Conflicts of Interest

The authors declare no conflicts of interest.

## Data Availability

The data that support the findings of this study are available from the corresponding author upon reasonable request.
